# Malignancies in patients with cystic fibrosis: a case series

**DOI:** 10.1186/s13256-021-03234-1

**Published:** 2022-01-19

**Authors:** Dorothea Appelt, Teresa Fuchs, Gratiana Steinkamp, Helmut Ellemunter

**Affiliations:** 1 Medical University of Innsbruck, Cystic Fibrosis Centre Innsbruck, 6020 Innsbruck, Austria; 2 Clinical Research and Medical Scientific Writing, Schwerin, Germany

**Keywords:** Cystic fibrosis, Mucoviscidosis, Cancer, Tumor, Case report

## Abstract

**Background:**

Previous reports have shown an increased number of colorectal cancers in patients with cystic fibrosis. We assessed the database of our cystic fibrosis center to identify patients with all kinds of cancer retrospectively. All patients visiting the Cystic Fibrosis Centre Innsbruck between 1995 and 2019 were included.

**Case presentation:**

Among 229 patients with cystic fibrosis treated at the Cystic Fibrosis Centre in Innsbruck between 1995 and 2019, 11 subjects were diagnosed with a malignant disease. The median age at diagnosis was 25.2 years (mean 24.3 years). There were four gynecological malignancies (cervical intraepithelial neoplasia and cervical cancer), two hematological malignancies (acute lymphocytic leukemia), one gastrointestinal malignancy (peritoneal mesothelioma), and four malignancies from other origins (malignant melanoma, neuroblastoma, adrenocortical carcinoma, and thyroid cancer). One malignancy occurred after lung transplantation. There was a strong preponderance of females, with 10 of the 11 cases occurring in women. Six deaths were attributed to cancer.

**Conclusions:**

Most diagnoses were made below 30 years of age, and half of the subjects died from the malignant disease. Awareness of a possible malignancy is needed in patients with atypical symptoms. Regular screenings for cancer should also be considered, not only for gastrointestinal tumors.

## Background

Improvements in the management of cystic fibrosis (CF) have resulted in better survival of patients, with increasing numbers of patients reaching adulthood. It also seems to be more common that patients with CF suffer from cancer [1, 2]. An increased risk of gastrointestinal cancers among CF patients is known [3–6], whereas other types of cancer have rarely been reported. A few studies showed differing results with similar [7, 8] or higher risk of cancer [3, 9] compared with non-CF cohorts. To assess the prevalence of malignant disease in our patients, we collected data from the patient database at the CF Centre Innsbruck from 1995 to 2019, including diagnosis, treatment, and outcome of the malignant condition. Among 229 patients with CF, we observed 11 cases with cancer over a period of 24 years.

## Case presentation

### Case 1

A 9-month-old Caucasion girl, who had been diagnosed with CF at the age of 1 month with an abnormal newborn screening, had a routine abdominal ultrasound, where a neuroblastoma stage 4s was diagnosed. At the time of diagnosis, no symptoms were present. The entire tumor was surgically removed, and she received chemotherapy according to the European HR-NBL-1/ESIOP protocol followed by an autologous bone-marrow stem-cell transplantation. At 6 years, the patient presented with pain in the left proximal tibia. Osteomyelitis was suspected, but antibiotic treatment showed no improvement in symptoms. The suspicion of a systemic relapse of the neuroblastoma was confirmed histologically. Chemotherapy according to protocol ESIOP TVD was started. After the fourth cycle, the tumor cells showed resistance to treatment and the disease progressed with changes in the bone marrow. Therapy was intensified with chemotherapy, radiation therapy, and allogeneic stem cell transplantation. Under this therapy, the patient was clinically stable with recurring aplasia, thrombocytopenia, and anemia. Just after her ninth birthday, she presented with a pulmonary exacerbation, which improved only after discontinuation of immunosuppression. The patient and her parents decided to continue with palliative therapy with fractionated low-dose 131I-metaiodobenzylguanidine. Three years after stem cell transplantation, the patient died at home surrounded by family.

### Case 2

A 14-year-old Caucasion girl with CF presented with fever, urticaria, joint pain, fatigue, and reduced general condition. She was diagnosed with c-ALL B-II and admitted for treatment. Chemotherapy was started under protocol AIEOP-BFM ALL 2000. Three weeks after diagnosis, she was discharged. Two days later, she presented with symptoms of a distal intestinal obstruction syndrome (DIOS) with constipation, weakness, hypoglycemia, and hypotonic dehydration. Her condition improved slightly after enema and antibiotic treatment, but she soon developed fever. Chest x-ray showed several peripheral infiltrates in the lungs, so antifungal therapy with amphotericin B was started according to local standard procedures. Despite decreasing inflammatory parameters, her general condition worsened with dyspnea, vertigo, and scintillating scotoma. A head CT scan showed seven brain abscesses. The girl died 1 month and 4 days after diagnosis of c-ALL. The autopsy showed endocarditis with septic abscesses in the brain, lungs, liver, kidney, and spleen. Microbiological examination of blood detected *Saccharomyces cerevisiae*, sensitive to itraconazole and resistant to amphotericin B.

### Case 3

A 17-year-old Caucasion girl with CF was admitted to the intensive care unit (ICU) with strong suspicion of leukemia. She was diagnosed with acute lymphocytic leukemia (ALL) B-II without central nervous system (CNS) involvement. She received chemotherapy under protocol AIEOP-BFM 2009. The ALL was classified as “intermediate risk.” For aplasia, the patient received antibiotic and antifungal prophylaxis and erythrocyte and platelet concentrates. She developed abdominal pain due to *Clostridium difficile*-associated colitis and considerable accumulation of ascites. With abdominal paracentesis, 6 L of ascites was removed. Then she developed constipation that did not improve with Macrogol therapy. Endoscopic stool removal was performed. Two months after beginning treatment, the patient had peritonitis with *Staphylococcus hominis*. Antibiotic treatment was started. Chemotherapy was discontinued due to the high risk of infection. She then developed hepatorenal syndrome with a known liver fibrosis and decreasing urine output. Intermittent hemodiafiltration and hemodialysis were necessary. In addition, the patient had deteriorating liver function values. A bone marrow biopsy showed no progression of the leukemia. Infection parameters increased nonetheless. There were multiple possible foci such as colitis with *Clostridium difficile*, peritonitis with *Staphylococcus hominis*, detection of atypical nontuberculosis mycobacteria in sputum, and a local infection of the central venous catheter. Two months and 9 days after diagnosis of ALL, the patient developed multiple organ dysfunction syndrome and died.

### Case 4

A 21-year-old Caucasion woman had an excision of a malign melanoma on her back. Histology showed a Breslow thickness of 1.9 mm and Clark level IV. A re-excision with 1 cm safety margin and a biopsy of sentinel lymph nodes were performed, showing no sign of metastases. Seven years after diagnosis of the melanoma, the patient remains tumor free.

### Case 5

A 22-year-old Caucasion woman presented with Cushing’s syndrome. Further investigations showed an adrenal carcinoma. The patient underwent surgery where the entire tumor was removed. The staging showed no metastases, so chemotherapy was not added to the treatment. Ten months after the first symptoms, the woman had a local tumor relapse with metastases in the lung, femur, and tibia. The tumor was unresectable, and the patient refused further chemotherapy. She started palliative therapy with radiation of the painful metastases in the femur and tibia. The disease progressed, and the patient showed psychological alterations. Airway clearance therapy became more difficult and less effective, and the respiratory status of the patient worsened. Fifteen months after diagnosis, the patient died due to respiratory insufficiency.

### Case 6

A 25-year-old Caucasion woman had an abnormal Pap smear (PAP IV) followed by a cervical conization and fractional abrasion. Histology showed a cervical glandular intraepithelial neoplasia (CGIN II) with a positive resection margin. The patient refused a hysterectomy at that time. The gynecological follow-ups with Pap smear and abrasion showed no residuum. Over the years, the pulmonary condition of the woman constantly declined with severe pulmonary hemorrhage at age 29. An angiographic coiling was performed that stabilized the condition for some time. She had to be ventilated and received extracorporeal membrane oxygenation but, despite an emergency lung transplantation, died due to organ failure.

### Case 7

A 28-year-old Caucasion woman with CF liver disease had a decompensation with increasing amounts of ascites. Diuretic therapy was unsatisfactory, so an ascites puncture was done. Laboratory investigation of the ascites showed a high cell count and high protein concentrations suggestive of an inflammatory cause with no sign of malignancy. In addition, the bacteriological cultures of ascites, blood, and urine were sterile. Only the sputum showed a known infection of *Pseudomonas aeruginosa*. An intravenous suppressive antibiotic treatment was started. A positron emission tomography–computed tomography (PET-CT) scan showed changes in the lungs consistent with CF and liver cirrhosis with portal hypertension and severe peritonitis with mesentery thickening.

The patient developed severe hypoglycemia and increasing anemia. Gastroscopy showed small esophageal varices without any sign of bleeding. She received intravenous glucose and erythrocyte concentrates. An X-ray of the lung showed growing consolidations in the inferior lobes of the lung. She was transferred to the intensive care unit. Hemofiltration was started because of increasing metabolic acidosis and anuria. The antibiotic treatment was adapted several times, but inflammatory parameters did not improve. No other focus except for the known pulmonary infection could be found. About 1 month after admission to hospital, the patient had more abdominal pain despite permanent analgesic therapy. A CT scan of the abdomen showed a toxic mega colon and a massive growth of the mesentery bulk. A fine-needle biopsy showed a malignant deciduoid mesothelioma. Based on the diagnosis, a multidisciplinary team suggested palliative care, to which the patient and her family agreed. Six weeks after admission to the hospital, the patient died.

### Case 8

On a screening examination in the 15th week of pregnancy, a 28-year-old Caucasion woman had an abnormal Pap smear (PAP IV). Cervical conization showed an adenocarcinoma that had a positive resection margin histologically. The patient had two more conizations, which had a positive resection margin again in histology, and a Shirodkar cerclage was placed. At 32 weeks of pregnancy, fetal lung maturity was induced and a healthy child was born via cesarean section. In the same operation, a radical Wertheim hysterectomy was performed. Histology showed an adenocarcinoma grade 2 with negative resection margins and micrometastases in one of 22 examined lymph nodes. Combined treatment with chemotherapy (cisplatin) and radiation was performed. Six years after diagnosis, the patient is still in remission.

### Case 9

Due to an abnormal Pap smear (PAP IV), a 31-year-old Caucasion woman had a cervical conization and fractional abrasion with CGIN grade II. All resection margins were negative. The patient then developed menometrorrhagia and dyspareunia. A hysterectomy was performed 4 years after diagnosis because of discomfort. The patient is still in remission 15 years after diagnosis.

### Case 10

A 36-year-old Caucasion woman presented with recurring vaginal bleeding. Further gynecological examination with a biopsy showed a cervix carcinoma grade II b. The histology after laparoscopic lymphadenectomy was tumor free. Two weeks after diagnosis, radiochemotherapy was initiated. Five months after diagnosis, a biopsy of the cervix showed mostly necrotic tumor tissue with 10% vital tissue. The woman had surgery with hysterectomy, salpingectomy, and iliac lymphadenectomy. Histology showed an invasive, low-differentiated, nonkeratinized squamous cell carcinoma of the cervix and three of five lymph nodes with metastases. After surgery, a PET-CT scan showed complete remission. Seven months after diagnosis, the woman presented with fever and pain in the groin. A CT scan showed a lymphocele that was punctured, streptococcus was detected, and treatment with antibiotics was started. The check-up examination 12 months after diagnosis with magnetic resonance imaging (MRI) and PET-CT scans showed an extensive vaginal stump relapse. Chemotherapy was started, but the disease progressed despite therapy. A multidisciplinary team suggested to continue with palliative care, to which the patient and her family agreed. Seventeen months after diagnosis, the patient died.

### Case 11

A 40-year-old Caucasion man with CF who had a lung transplant at 23, was admitted with a multinodular goiter for an operation. During the operation, an immediately frozen section showed bilateral papillary thyroid cancer (right 0.9 cm, left 1 cm). A total thyroidectomy with excision of local lymph nodes was performed. After the operation, a PET-CT-scan showed multiple glucose-metabolizing lesions in lymph nodes from neck to mediastinum. A neck dissection was performed. Histology showed multiple metastases in the lymph nodes. Thyroid hormones as suppression therapy were administered. Three months after diagnosis, therapy with radioactive iodine was started. In total, the man received five cycles with radioactive iodine. Five years after diagnosis, the patient still has a stable disease. The last PET-CT scan showed no glucose-metabolizing lesions.

**Table:** Malignancies in patients with Cystic Fibrosis at the CF Centre Innsbruck

Site of malignancy	Cases (*n*)	Sex (female:male)	Age at tumor diagnosis (years, mean [min–max])	Outcome (*n* complete remission:death)	CFTR variant (phe508del homozygous: other)	ppFEV_1_ before diagnosis (%, mean [min–max])
CIN/cervix carcinoma	4	4:0	30.6 [25.2–36.9]	3:1	3:1	79.8 [42.4–122.2]
Peritoneal mesothelioma	1	1:0	28.7	0:1	0:1	56.3
Acute lymphocytic leukemia	2	2:0	15.9 [14.7–17.2]	0:2	0:2	100.8 [99.3–102.4]
Malignant melanoma	1	1:0	21.5	1:0	1:0	91
Neuroblastoma	1	1:0	0.8	0:1	0:1	–
Adrenal carcinoma	1	1:0	22.7	0:1	0:1	43.8
Thyroid cancer	1	0:1	40.2	1:0	1:0	75.3
All	11	10:1	24.3 [0.8–40.2]	5:6	5:6	78.6 [42.4–122.2]

*n* number, *CFTR* cystic fibrosis transmembrane regulator, *ppFEV1* percent predicted forced expiratory volume in one second, *CIN* Cervical intraepithelial neoplasia

In total, we report ten female patients and one male patient with CF and cancer. The patients had a mean age of 24.3 years, about half of the patients were phe508del homozygous, and more than half had died by the end of our observation. The mean of the most recent lung function before cancer diagnosis showed a forced expiratory volume in one second (FEV_1_) of 78.6% of predicted normal value.

## Discussion

Among 229 patients with cystic fibrosis visiting our CF Centre between 1995 and 2019, 11 cases of cancer were diagnosed, mainly in the third decade of life. Ten patients were female, four of whom had cervical cancer or cervical intraepithelial neoplasia. Six women died from cancer. Cancer screening could potentially prevent early deaths and should be included in routine diagnostics for adults with cystic fibrosis.

This is the largest case series of CF patients with cancer diagnoses. Previous reports show that patients with CF carry an increased risk for gastrointestinal cancer, and they appear to have an earlier onset of cancer than otherwise healthy adults [6, 10–15]. The patients in our case series had a mean age of only 24.3 years at cancer diagnosis. For cervical cancer, the average age at diagnosis worldwide in 2018 was 53 years, while our four patients were much younger when diagnosed at 25–39 years of age [16].

The lung function of the patients before malignancy diagnosis was mostly normal despite cystic fibrosis, with a mean forced expiratory volume in one second (FEV_1_) of 78.6 % of the predicted normal value. According to recent European CF Registry data, mean FEV_1_ % predicted for patients aged 18 years or older without a transplant is 68.5% [17]. Only one death (case 6) was associated with severe CF lung disease and pulmonary hemorrhage. Thus, cancer shortened life considerably in half of the patients.

The Cystic Fibrosis Foundation recommends to start colonoscopy as a screening for colorectal cancer in patients with CF starting at the age of 40 years [18]. In our patient group, only one patient was diagnosed with gastrointestinal cancer (peritoneal mesothelioma, case 7), while more patients had gynecological or hematological malignancies. The low number of colorectal cancers in our subgroup may be due to the screening programme at our center, with colonoscopy and therapeutic polypectomy on a regular basis starting at the age of 40 years.

From a total of 11 cases, we report 4 cases of cervical cancer or cervical intraepithelial neoplasia in 229 CF patients over a period of 24 years (mean age 30.6 years). This corresponds to a rate of 0.073% of patients per year. The incidence rate of cervical cancer per year (mean of 1995–2018) in the general population in Tyrol, Austria, where the CF Centre Innsbruck is located, is 0.0015% for the same age range [19]. This is a 49-times-higher incidence of cervical cancer in our collective compared with the general population in the same region for the same age range. Significant expression of cystic fibrosis transmembrane conductance regulator (CFTR) gene in the cervical epithelium has been reported [20]. An analysis of Pap smear tests in women with CF also showed a high proportion of abnormal tests and cervical dysplasia [21]. Considerably higher expression of CFTR in ovarian cancer was seen *in vitro* and *in vivo*, so downregulation of CFTR or dysfunctional CFTR should suppress aggressive malignant biological behaviors of ovarian cancer cells [22]. However, compared with cells from normal endometrium, the expression of CFTR was significantly upregulated in endometrial carcinoma cells, which could result in increased proliferation and transfer of endometrial carcinoma cells [23]. Due to CFTR dysfunction in the cervix, women with CF have an abnormally thick and dense cervical mucus [24]. The squamocolumnar junction is prone to human papillomavirus (HPV) infection and cell dysfunction [24].

The CFTR plays a role in multiple cellular processes, such as development, epithelial differentiation and polarization, regeneration, migration, proliferation, and epithelial–mesenchymal transition. Several studies suggest that CFTR exerts variable effects in different tissues and in different cancer types. Especially downregulation of CFTR seems to lead to tumorigenesis and invasiveness of tumors, but the exact mechanisms are still unknown [25]. The correlation between CF and a higher risk for gastrointestinal cancer may be explained by chronic inflammation, dysmotility, and altered fecal microbiome in the gut [7, 15, 26].

Another potential risk factor for cancers in cystic fibrosis is transplantation. After organ replacement, CF patients were shown to have an overall increased risk of cancer and specifically an elevated risk of gastrointestinal malignancy and lymphoma. This has largely been attributed to the intense, long-term immunosuppressive medication [5]. In our case series, only 1 of 11 patients had received a lung transplant.

Frequent exposure to radiation could be an additional reason for increased cancer risk. In most centers, patients receive chest X-rays on a yearly basis and further imaging in case of pulmonary exacerbations. At our center, we perform ultra-low-dose high-resolution chest tomography to document any progression of lung disease. Nevertheless, we are very aware of the fact that routine use of computed tomography needs to be done with great care for future implications [27]. In the future, CT may be replaced by MRI imaging for monitoring of pulmonary structural changes and inflammation [28].

Only a few studies record the occurrence of cancer in patients with CF over a longer period of time, and through our database we had access to 24 years of patient data. However, this is a retrospective study with a small patient collective, during a time where the treatment of CF is constantly developing. Since the association of some cancer types such as gastrointestinal cancer with CF is clear, other cancers such as neuroblastoma or leukemia may appear coincidental. No association between neuroblastoma and CF is known; only three cases of CF and neuroblastoma have been reported in literature [29, 30], and no causality is assumed.

## Conclusions

Further studies about cancer risk in patients with CF and a larger patient collective are needed. Ideally, the data could be obtained from national patient registries. Improvement of life expectancy in patients with CF increases the expected absolute risk of malignancies; therefore, it is necessary to integrate screening in the care of patients with CF.

Our data suggest an increased risk for gynecological malignancies. Considering the role of CFTR in endometrial carcinoma cells [31] and in the cervical epithelium, intensified screening for women with CF, compared with healthy women, may be reasonable, as well as routine HPV vaccination. In light of this, we recommend screening for gastrointestinal and gynecological malignancies. Special alertness for malignant diseases is obviously needed in patients after transplantation due to long-term immunosuppression. Screening for other types of malignant tumors should be included in the regular follow-up of adult patients. The role of CFTR in risk of cancer may change considering the effect of new CFTR modulator therapies on CFTR, and this needs to be investigated in further studies.

## Data Availability

The datasets analyzed during the current study are available from the corresponding author on reasonable request.

## Abbreviations

ALL: Acute lymphocytic leukemia
c-ALL B-II: Common acute lymphoblastic leucemia Type II B
CF: Cystic fibrosis
CFTR: Cystic fibrosis transmembrane conductance regulator
CGIN: Cervical glandular intraepithelial neoplasia
CNS: Central nervous system
CT: Computer tomography
DIOS: Distal intestinal obstruction syndrome
HPV: Human papillomavirus
ICU: Intensive care unit
MRI: Magnetic resonance imaging
PET: Positron emission tomography
